# On Intra-Uterine Injections in Hemorrhage

**Published:** 1874-09-15

**Authors:** William Draper

**Affiliations:** M. R. C. S., &c., York. Formerly Resident Obstetric Officer to the Middlesex Hospital


### Gleanings from Our Exchanges.

### ON INTRA-UTERINE INJECTIONS IN HEMORRHAGE.

By William Draper, M. R. C. S., &c., York. Formerly Resident Obstetric Officer to the Middlesex Hospital.

*From the Obstetrical Journal of Great Britain and Ireland.*

WITH the view of adding to the evidence concerning a subject so important as that of injecting fluid styptics into the uterus in hemorrhage from that organ, I publish a short summary of my observations during the seven years which I have practised the treatment. I much regret that I have not preserved any record of the post-partum hemorrhage cases in which I have employed injections of the solution of perchloride of iron; yet, although I have used this form of injection of various strengths, in a considerable number of cases, I can confidently say that, personally, I have *never* met with an instance in which such injections into the uterine cavity have done harm, and rarely with one in which they have failed to do good; indeed, most commonly have I found their use of the most decided and prompt benefit.

Serious uterine hemorrhage must always be looked upon as a grave matter, even by the most experienced practioners; still, in such alarming conditions, I now certainly find not a little solace and confidence in the feeling that, should the ordinary means to arrest the flooding fail, there is still a *dernier ressort* in intra-uterine injection, which is almost certain to bring the case to a favorable issue, if timely use of it be made.

In forms of uterine hemorrhage other than post-partum, such, for instance, as profuse menorrhagia, I have employed intra-uterine injections, not only of solutions of iron, but also of tannic acid, infusion of matico, and iced water. I append three cases illustrating different forms of hemorrhage in which some of these fluids have been injected with beneficial results.

As a rule I am of opinion that the iron injections are the most reliable, still I think there are certain conditions (as in Case I.), in which some of the other styptics might perhaps be more suitable.

Case I.—I was called to this case by a patient who was in the eighth month of pregnancy; for three or four days before consulting me, she had suffered very considerable hemorrhage. A vaginal examination discovered the cervix uteri soft and yielding, and about two-thirds obliterated. The tip of the index finger passed freely into the external os uteri; but the internal os was not dilatable. I ordered the patient to remain in bed, to have a draught containing sulphuric acid and laudanum every three hours, with cold to the vulva, and applied a firm abdominal bandage. In the evening of the same day the hemorrhage became still more violent. The external os now admitted the finger freely. On passing my hand into the vagina, I was enabled to reach the internal os, which, with difficulty, admitted the finger end, but which appeared dilatable. The fetal head could be felt presenting, but nothing like placenta was discovered. The hemorrhage being really alarming, I passed an elastic catheter into the uterus (carefully avoiding the membranes), and injected about two ounces of a strong infusion of matico, and then plugged the vagina. The patient being much exhausted, beef-tea and brandy were given freely. There were no labor pains. The following morning, when I removed the plug, some slight hemorrhage occurred, so the uterus was again injected with the matico infusion, and the vagina replugged. There was rather more dilatation of the os. When the plug was again removed there was no recurrence of hemorrhage. Labor set in naturally some days later, and came to a favourable termination, a living and healthy child being the result.

Case II. illustrates the successful employment of iced water as an intra-uterine injection. I was called to this case some time after the expulsion of the placenta, the labor having been a natural one. I found the patient much exhausted from the loss she had sustained, and still flooding violently. Having restored her somewhat with brandy, etc., I removed some large coagula from the uterus, gave ergot, applied pressure, &c.; in fact, I employed all the ordinary means to arrest the flooding without avail. I then passed a large gum-elastic catheter (having a syringe attached), into the cavity of the uterus, and injected several ounces of iced water. The uterus almost immediately contracted; pressure then being applied, the contraction was *kept* up, and no further hemorrhage occurred.

Case III.—Some time since a lady came under my care, whom I ascertained to be suffering from retroversion of the uterus. One of her most troublesome symptoms was very profuse and frequent menstruation, which was present, to a very serious extent, at the time she consulted me. Almost every available remedy was prescribed without having any influence over the discharge, and as the patient was reduced to an exceedingly weak state, I resolved to inject the uterus with solution of iron. Accordingly, I injected into the uterine cavity about two ounces of a solution of tincture of perchloride of iron, of the strength of one part to ten parts of water.

The hemorrhage was at once arrested, and there was no recurrence of it, nor did the slightest unfavorable symptom follow the practice. A Hodge's pessary was now applied, and, so long as it was worn, the menstrual periods passed over naturally, without either excessive discharge or pain. On one or two occasions, however, when the patient very injudiciously left off the pessary, serious menorrhagia recurred, and again the only successful means of arresting it was the iron injection, which never failed in its action, nor was it ever productive of the slightest untoward result.

